# Computer Vision and Augmented Reality for Human-Centered Fatigue Crack Inspection

**DOI:** 10.3390/s24113685

**Published:** 2024-06-06

**Authors:** Rushil Mojidra, Jian Li, Ali Mohammadkhorasani, Fernando Moreu, Caroline Bennett, William Collins

**Affiliations:** 1 Department of Civil, Environmental and Architectural Engineering, The University of Kansas, Lawrence, KS 66045, USA; rmojidra@ku.edu (R.M.); crb@ku.edu (C.B.); william.collins@ku.edu (W.C.); 2 Department of Electrical Engineering and Computer Science, The University of Kansas, Lawrence, KS 66045, USA; 3 Department of Civil, Construction and Environmental Engineering, University of New Mexico, Albuquerque, NM 87131, USA; amohammadkhorasan@unm.edu (A.M.); fmoreu@unm.edu (F.M.)

**Keywords:** fatigue cracks, computer vision, augmented reality, human-centered inspection, HoloLens2, bridge inspection

## Abstract

A significant percentage of bridges in the United States are serving beyond their 50-year design life, and many of them are in poor condition, making them vulnerable to fatigue cracks that can result in catastrophic failure. However, current fatigue crack inspection practice based on human vision is time-consuming, labor intensive, and prone to error. We present a novel human-centered bridge inspection methodology to enhance the efficiency and accuracy of fatigue crack detection by employing advanced technologies including computer vision and augmented reality (AR). In particular, a computer vision-based algorithm is developed to enable near-real-time fatigue crack detection by analyzing structural surface motion in a short video recorded by a moving camera of the AR headset. The approach monitors structural surfaces by tracking feature points and measuring variations in distances between feature point pairs to recognize the motion pattern associated with the crack opening and closing. Measuring distance changes between feature points, as opposed to their displacement changes before this improvement, eliminates the need of camera motion compensation and enables reliable and computationally efficient fatigue crack detection using the nonstationary AR headset. In addition, an AR environment is created and integrated with the computer vision algorithm. The crack detection results are transmitted to the AR headset worn by the bridge inspector, where they are converted into holograms and anchored on the bridge surface in the 3D real-world environment. The AR environment also provides virtual menus to support human-in-the-loop decision-making to determine optimal crack detection parameters. This human-centered approach with improved visualization and human–machine collaboration aids the inspector in making well-informed decisions in the field in a near-real-time fashion. The proposed crack detection method is comprehensively assessed using two laboratory test setups for both in-plane and out-of-plane fatigue cracks. Finally, using the integrated AR environment, a human-centered bridge inspection is conducted to demonstrate the efficacy and potential of the proposed methodology.

## 1. Introduction

Current practices in steel bridge fatigue crack inspection primarily relies on human vision. In the United States, to ensure inspection quality, the Federal Highway Administration (FHWA) and state departments of transportation have established guidelines concerning inspector qualifications, training, and certification. Nevertheless, Campbell et al. [1] assessed inspectors’ ability to detect fatigue cracks by inviting a diverse group of 30 inspectors to conduct hands-on inspections of 147 steel bridge specimens with known fatigue cracks. The crack lengths for an average of 50% and 90% detection rates were 26 mm and 113 mm, respectively, highlighting the limitations of visual inspection even under optimal conditions. Moreover, 42% of the bridges in the U.S. are at least 50 years old, and many of them are in poor condition [2]. In particular, fatigue cracks can lead to catastrophic failures in these bridges [3,4]. No existing fatigue crack detection techniques, whether contact-based or non-contact-based, can fully substitute human visual inspection with absolute accuracy and reliability. The involvement of human judgment remains essential for decision-making in the real-life bridge inspection process. Therefore, a human-in-the-loop approach, combining the expertise of the inspector with the capabilities of technology, can potentially result in better accuracy and efficiency in detecting fatigue cracks, thereby enhancing the safety and integrity of steel bridges.

Augmented reality (AR) is a great example of such technology that can provide inspectors with additional information and visual cues to aid in the detection and assessment of fatigue cracks. Azuma [5] defines AR as systems with three essential characteristics as follows: (1) it combines the real and virtual worlds by overlaying virtual elements onto the physical environment, (2) it provides real-time interaction between the virtual and real world, and (3) the virtual objectives are registered in three dimensions. Examples of AR devices include Microsoft’s HoloLens 1 (HL1), HoloLens 2 (HL2), and Magic Leap. Moreu et al. [6] used HL1 for a hands-free measurement of distance between two points on a structure. Napolitano et al. [7] demonstrated a system for documenting, visualizing and annotating data using an AR environment. Maharajan et al. [8] used an AR head-mounted device to access real-time data during a field inspection to enhance the field inspection. In an experimental validation, the bridge inspector visualized strain changes which helped the inspector make real-time decision based on changes in the surrounding environment. Kamat and El-Tawil [9] superimposed computer-aided design (CAD) shear wall images onto the actual structure using an AR device integrated with a GPS-based tracking system. Inter-story drift was measured by interpreting key differences between the real and augmented views of the facility. Dunston and Shin [10] applied AR to evaluate steel column tilt in a laboratory setting and reported higher efficiency than traditional manual inspection methods. Although AR devices can capture high-dimensional data such as images and videos, their computational power is still limited, making it challenging to provide near-real-time analysis results in many cases. To address the challenge, AR devices can leverage more computational power and analysis resources through wireless connection, allowing image and video data to be processed in real-time. The analysis results can then be sent back to the AR device, providing the inspector with necessary information for decision-making. Using this approach, Mohammadkhorasani et al. [11] developed an AR software integrating the computer vision-based fatigue crack detection algorithm by Mojidra et al. [12]. However, the algorithm itself lacks sufficient computational efficiency for nonstationary cameras, and the developed AR environment lacks the capability of enabling in-depth human–machine interaction for refining the crack detection result, as will be elaborated on later in the paper. 

Various image processing-based crack detection methods have been developed as a result of the recent rapid advancement in computer vision. Abdel-Qader et al. [13] used fast Haar transform, fast Fourier transform, and Sobel and Canny filters to detect cracks in concrete bridge images. Nishikawa et al. [14] developed multi-sequential filters to remove noise and detect cracks in concrete structures. Yamaguchi et al. [15] designed a percolation model to extract a continuous texture representing a crack depending on a length criterion. Iyer and Sinha [16] generated crack segmentation maps in pipe structure images by enhancing the contrast of input images, applying filters, and evaluating curvature in the cross direction to produce the final binary map for segmented cracks. However, these methods based on traditional image processing struggle to differentiate between real cracks and crack-like features such as edges, corrosion marks, and scratches. Recent advancements in artificial intelligence (AI) have led to the development of convolutional neural networks (CNNs), which are capable of extracting valuable information from images. For example, Cha and Choi [17] successfully detected cracks in concrete images under various challenging conditions, such as different lighting, shadows, blurs, and close-up views, by training a CNN for image analysis. 

The aforementioned techniques were designed primarily to detect cracks in concrete structures or pavements, which are relatively easier to detect compared to fatigue cracks in steel structures. This is because concrete cracks are typically more conspicuous and visually evident due to their wider and more irregular nature, while fatigue cracks in steel structures are often more subtle and harder to detect visually. In addition, images of steel structures often contain features that resemble cracks, such as corrosion marks, bolts, and bolt holes, which can make crack detection more challenging. Dung et al. [18] trained a CNN to identify fatigue cracks in steel bridges. The dataset images were partitioned into smaller segments to eliminate features resembling cracks in the segments containing actual cracks. Dong et al. [19] suggested a neural network with an encoder–decoder architecture, adapted from the U-Net structure, for segmenting fatigue crack pixels. However, the fatigue cracks were annotated by markers above the cracks, which made the detection easier. The authors observed that the network also identified marker curves, weld line edges, and handwriting as crack pixels. The performance of CNNs is often dependent on the quality and characteristics of the data used for training. Specifically, if the test images have significant variations or differences from the images used in the training dataset, it can lead to inaccurate or false predictions. To develop a neural network that can accurately detect fatigue cracks in a variety of bridge images, a significant amount of data gathering and labeling is required, leading to a process that is time-consuming and expensive. Furthermore, the issue of false positive predictions of other crack-like features remains a significant challenge in the development of accurate fatigue crack detection techniques.

To overcome the challenges associated with fatigue crack detection, Kong and Li [20] developed an approach through the use of a short video stream under live load for structural surface displacement tracking and analysis to accurately detect fatigue cracks. Specifically, a short video is recorded using a fixed camera to capture a region of interest (ROI) undergoing several fatigue cycles. Subsequently, feature points are identified to track the surface displacements, from which fatigue cracks are detected by recognizing differential displacement patterns caused by the crack opening and closing under fatigue load, which surpass a predefined threshold. The suggested method demonstrates strong robustness in the presence of crack-like features such as corrosion marks, dust, and bolt holes, as it relies not only on spatial surface features but also on temporal changes in these features. Unlike crack-like features that remain relatively stable over short periods, feature points around fatigue cracks experience substantial displacements due to the opening and closing of the cracks. Later, Mojidra et al. [12] improved the above displacement-based method for nonstationary cameras by introducing Global Motion Compensation (GMC) techniques specifically designed for both 2D and 3D videos. GMC eliminates camera motion from the video stream, such that the tracked displacements are free of the camera motion and the same method by Kong and Li [20] can be applied to videos captured by cameras hosted on a moving platform such as unmanned aerial vehicles and wearable devices. Nonetheless, a limitation of the displacement-based crack detection method is its dependence on GMC, which is computationally expensive. For example, the authors reported that the GMC took 1.1 s per video frame for 2D videos, while for 3D videos with reduced resolution, it cost 1.75 s per frame. A 5–6 s video with 30 frames per second could take up to 5 min for processing, posing a challenge for achieving near-real-time human-centered bridge inspection. 

In this paper, we present a novel human-centered fatigue crack inspection methodology empowered by AR and computer vision. The novelties and contributions of this paper are twofold as follows: (1) improvement of the crack detection algorithm by Mojidra et al. [12] and development of a method to quantify the detection accuracy and (2) integration of the algorithm into an AR environment with unique features to enable human–machine interaction for bridge inspections. First, for fatigue crack detection using a moving camera, we further improved the video-based method [12] by eliminating the need for GMC, achieving near-real-time results with higher accuracy. Rather than analyzing the absolute displacements of feature points, we switch to tracking the distance change between feature point pairs, which effectively removed the impact of any rigid body motion including those induced by camera movement, eliminating the need for GMC. In addition, unlike other methods, our crack detection result is composed of discrete feature points surrounding the crack. No method currently exists to quantify its accuracy. To bridge this gap, this paper proposes a new method to quantify the crack detection result through clustering analysis. Furthermore, an interactive AR environment is created to integrate the crack detection algorithm and facilitate human–machine interaction for achieving optimal detection results. The AR environment enables the inspector to perform video acquisition via the embedded camera of the AR headset, then sends the video to the cloud server for processing. The processing results are then converted into holograms. In particular, these holograms, along with an AR virtual menu that allows the inspector to toggle through various crack detection parameters, enable in-depth human–machine interaction to refine the crack detection results, hence facilitating human-in-the-loop decision-making. Finally, the integrated human-centered bridge inspection process is demonstrated using a laboratory bridge specimen through near real-time crack detection.

## 2. Methodology

**Figure 1:**
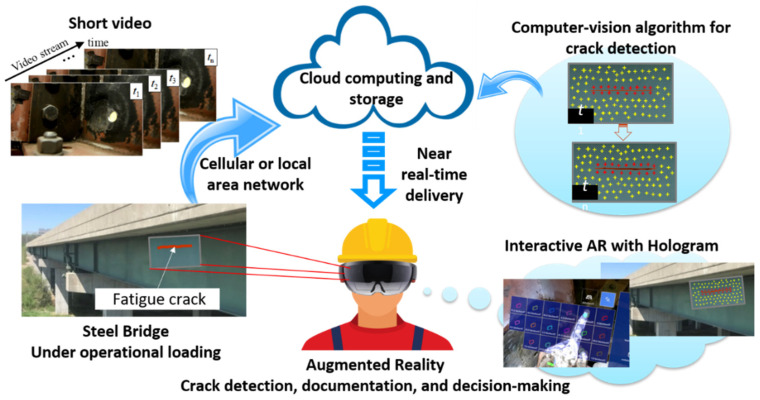
The proposed human-centered bridge inspection process empowered by AR and computer vision.

This section describes the methodology of the proposed human-centered bridge inspection process. Figure 1 depicts the overall concept and the essential components. First, a local area network (LAN) is created using a Wi-Fi router, which establishes a connection between the AR headset, e.g., HL2, and the server computer which is responsible for performing the developed crack detection algorithm and data storage (raw video data and processing results). Then, the bridge inspector proceeds to record a short video of a fatigue-crack-prone region in the structure to capture several fatigue load cycles using the embedded camera through the developed AR application. The video is then automatically transmitted to the server through the LAN connection, where the video is stored and processed for crack detection. Note that the LAN can be replaced by a cellular network, which connects to a remote server computer for data processing and storage. 

To process the video at the server, an ROI is first selected based on the first video frame. Distinctive feature points, determined by pixel intensity gradient of the image, are identified in the first frame. Their positions are then tracked throughout the video. Subsequently, these features are grouped into small local circular regions (LCRs), within which the distance between each feature point pair is calculated. Feature point pairs not situated on opposite sides of the fatigue crack display minimal distance changes. Otherwise, they exhibit distance variations resulting from the crack opening and closing. By examining the distance history of all unique feature point pairs within the LCRs and identifying significant distance changes that surpass a threshold value, the feature pairs associated with a fatigue crack within the LCRs are isolated and highlighted. Ultimately, the collection of highlighted feature points, or feature point cluster, outlines the location of the fatigue crack. Compared to the method in [12], which performs GMC first before tracking the absolute displacements, this improved method does not require GMC for removing camera motion as tracking distance changes effectively eliminates the impact of rigid-body motions, hence it possesses much higher computational efficiency and generates results in near-real-time. A more in-depth explanation of the improved crack detection algorithm is provided in the subsequent section.

Further, since the optimal threshold value cannot be uniquely defined for all situations, human input in the field is essential to ensure success. To facilitate in-depth human–machine interaction, a range of threshold values are applied in the algorithm at the server to produce a group of feature point clusters as candidate crack detection results, which are then transmitted to the AR device via the LAN. The AR application converts each feature point cluster into a hologram which can be overlaid onto the real-world view of the actual structure for further examination. Meanwhile, an interactive virtual menu is presented to the inspector, in which the threshold values can be selected to view the associated crack detection results. By toggling through the range of threshold values, the inspector interacts with the holograms and decides the optimal threshold for the final crack detection result. This enhanced visualization and visual indicators help the inspector in detecting fatigue cracks that may be imperceptible to the naked eye, enabling them to make more informed decisions during the inspection process.

## 3. Crack Detection Algorithm

The core principle of the proposed crack detection approach is adopted from [20], which focuses on identifying crack-induced surface motion under fatigue loading, rather than merely extracting crack edges and features. The recorded video is analyzed to detect discontinuities caused by fatigue crack opening and closing under live load. Feature points, which are specific pixels in images with high intensity gradient, are detected and used to track the surface motion. Notable algorithms for feature point detection include Harris–Stephens [21], Scale Invariant Feature Transform (SIFT) [22], Speeded-Up Robust Features (SURF) [23], Shi–Tomasi [24], and Features from Accelerated Segment Test (FAST) [25]. In this study, the Shi–Tomasi algorithm was chosen for its robust performance because it is improved upon the Harris–Stephens method by using the minimum eigenvalue of the gradient matrix for corner detection, leading to more accurate and reliable feature point detection in many scenarios. Moreover, the Shi–Tomasi algorithm is relatively simple and computationally efficient compared to more complex methods like SIFT and SURF. This makes it suitable for real-time applications where speed is crucial. Feature points are detected within the selected ROI in the first frame of the video. These features are then tracked in subsequent video frames using the Kanade–Lucas–Tomasi (KLT) tracker [26,27]. For robust tracking, we implemented forward and backward tracking [28] as well as five pyramid level representations in the KLT tracker. One advantage of using the KLT tracker is its computation efficiency, as it is based on sparse optical flow. 

However, the original method tracks absolute displacements of surface motion and only works for videos taken by a fixed camera. Mojidra et al. [12] introduced GMC to remove the camera motion, but it significantly increased the processing time. In this section, to achieve accurate detection results while minimizing computation, a method is proposed based on tracking distance change between feature point pairs. 

**Figure 2:**
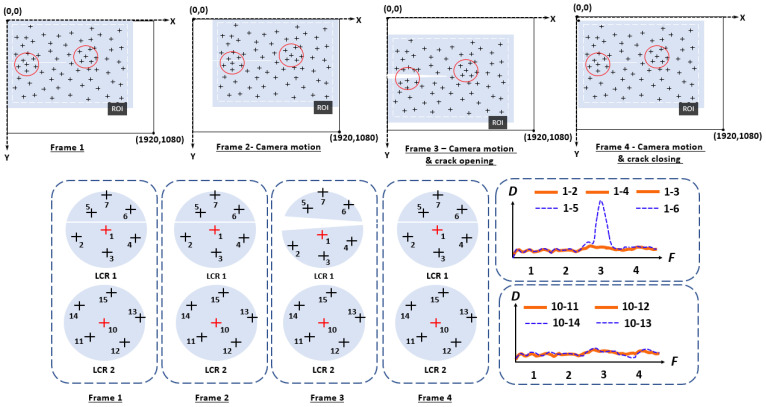
Illustration of the proposed crack detection algorithm based on surface distance tracking.

To illustrate the distance-based crack detection algorithm, consider the four video frames from a video stream as shown in Figure 2. The camera’s field of view, represented by the black rectangle, covers a steel plate (illustrated by the blue rectangle) and other background elements. The detected feature points in the selected ROI are indicated by the plus symbols. Two LCRs are evaluated including LCR 1, situated over the fatigue crack, and LCR 2, located away from the fatigue crack. LCR 1 contains a total of seven feature points, with feature point 1 at the center of the LCR. Feature points 1 to 4 are beneath the fatigue crack, while points 5 to 7 are above the crack. Meanwhile, LCR 2 encompasses a total of six feature points, with feature point 10 situated at the center of the LCR. As an illustration, in Frame 2, due to the camera movement, the steel plate has moved to the right within the camera’s field of view. In Frame 3, the camera has moved further, and the fatigue crack has opened under the live load. In Frame 4, the steel plate has moved upward, and the fatigue crack has closed. The distances between the central feature point and the remaining feature points within the same LCRs of all four video frames are depicted at the bottom right of Figure 2.

The distance between feature point pairs within the LCRs in the first and second frames remains virtually identical because the global camera motion only introduces ridge-body movements, hence feature points within a local vicinity move in an identical manner. However, in Frame 3 and Frame 4, the distances between the feature point pairs that cross the crack have increased due to the crack opening or closing, and the distance between the remaining feature pairs that do not cross indicate a crack that continues to remain unchanged. In this case, LCR 1 contains two different patterns in the distances among feature points due to the crack’s response to live load, whereas LCR 2, lacking crack activity, shows only one pattern. The presence of camera-induced rigid body motion did not influence distance patterns in both cases, eliminating the requirement for motion compensation when distance serves as the metric. Consequently, the computationally demanding process to remove the camera motion, a crucial part of the displacement-based method [12], becomes unnecessary for the distance-based method, leading to much improved computation efficiency, which is essential for near-real-time crack detection for field applications. Moreover, as will be described in the validation studies, this distance-based method also outperforms the displacement-based approach in terms of crack detection accuracy. 

To effectively analyze the entire ROI, an initial random feature point is chosen as the center for the LCR, followed by a search to collect feature points within the LCR. Next, the standard deviations of distance histories for all unique feature pairs are computed. If the standard deviation of the distance history falls below a certain threshold value, no differential motion is detected. Conversely, if the standard deviation exceeds the threshold value, the feature point pair is highlighted in the results. This process is repeated by selecting a different feature point as the center of the next LCR until all feature points have been analyzed. The final crack detection result comprises clusters of all the highlighted feature points for which a differential movement pattern is identified within the associated LCR. As these highlighted feature point clusters trace along the fatigue crack, they intuitively indicate the location and extent of the detected crack. As explained previously, a range of threshold values are employed to filter out feature point pairs with low distance variations, generating a series of outputs. Subsequently, the inspector can toggle through the holograms of this series of feature point clusters as the crack detection results at the given bridge size, and the enhanced visualization aids in decision-making in terms of selecting the optimal threshold.

## 4. AR Environment

An AR software package has been developed to create a holographic interface and facilitate human-centered bridge inspections. We utilized the HL2 as the AR device, which is capable of performing 3D scanning (spatial mapping) of the surrounding environment, programming, and projection. A server, which could be a laptop or PC, runs both a crack detection algorithm in MATLAB (R2021a) [29] and a Structured Query Language (SQL) database. This setup facilitates communication with an HL2 device through a local area network (LAN), established using a wireless router that also serves as a Wi-Fi hotspot. The network allows for bidirectional communication, enabling the SQL database on the server to send and receive data not only with the HL2 but also with the MATLAB application. For robust and seamless interaction between the HL2 and the server, it is crucial that both devices remain within the range of the Wi-Fi hotspot. The database transmits the video captured by the HL2 to the server for crack detection analysis, and the results are sent back to the HL2 to generate a hologram. An Auto Anchoring System (AAS) has also been developed for automatic anchoring of holograms onto the structure. More details on the AAS and the database are described in [11,30].

**Figure 3:**
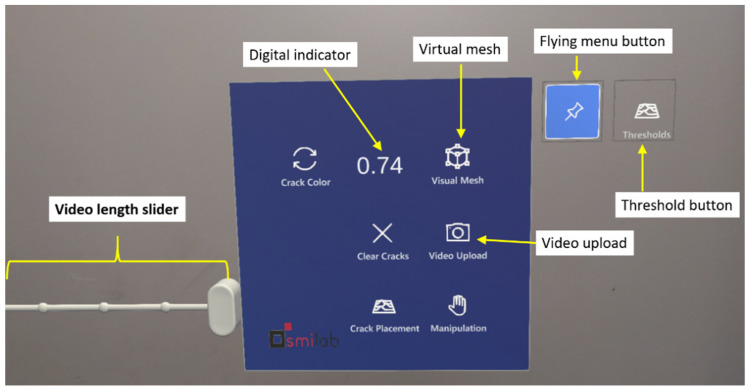
The main virtual menu of the developed AR environment.

**Figure 4:**
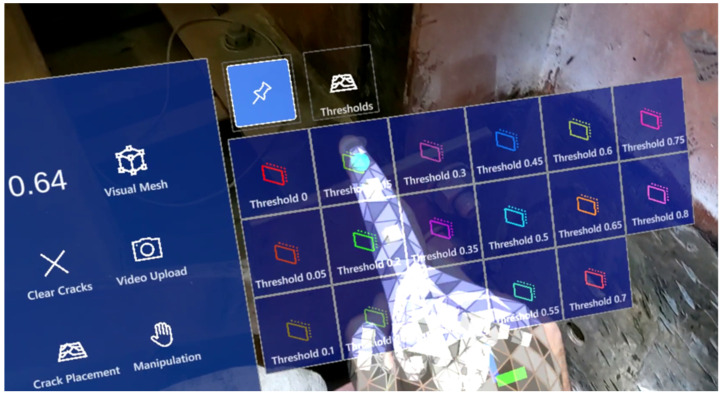
Virtual menu with threshold options for human-in-the-loop decision-making.

To provide a user-friendly AR interface, a virtual menu has been designed for the AR software, enabling users to smoothly operate and interact with the software. Figure 3 displays the virtual menu with its associated functionalities labeled. A “Visual Mesh” button can be found on the top right section of the virtual menu. The visual mesh button leverages HL2’s spatial mapping capabilities, which involve its comprehension of the surrounding environment. This feature allows the user to visualize all objects that the HL2 assesses as part of its 3D surroundings, enabling the inspector to confirm that the HL2 is examining the correct surface during the inspection. The video upload button facilitates video recording during the inspection process. The slider for controlling video length allows the user to adjust the video duration (between 1 and 10 s) captured by the HL2. This feature enables users to ensure that an adequate number of fatigue load cycles are included in the recorded video. The flying menu button toggles the virtual menu between flying and hovering modes. The flying mode enables the menu to follow the inspector’s movement during the inspection, freeing the inspector’s hands for other tasks and eliminating the need to manually move the menu. In contrast, the hovering mode is suitable when the inspector needs to inspect a specific section of the structure. By switching to the hovering mode, the inspector can manually reposition the virtual menu as needed, allowing for more focused examination. The “Thresholds” function presents a series of virtual buttons corresponding to the number of thresholds for crack detection specified by the inspector. As illustrated in Figure 4, the virtual threshold options are displayed upon pressing the “Thresholds” button. Each button represents a single threshold result produced by the crack detection algorithm. This feature is the key to enabling human-in-the-loop decision-making, allowing the inspector to interact with the crack detection results, effortlessly reviewing the outcomes associated with a range of thresholds, thereby enabling the selection of the most appropriate result for documentation, monitoring, and informed decision-making.

## 5. Experimental Setups

The proposed distance-based crack detection method was experimentally tested using the same laboratory setups as the displacement-based method [12] through both 2D and 3D videos. A scene can be classified as 2D if it lies on a flat plane, is significantly far from the camera, or has minor variations in distance from the camera compared to the overall camera-to-scene distance. In contrast, a 3D scene involves greater depth and more noticeable distance variations relative to the camera position. More details and discussions on 2D and 3D videos are provided in [12], from which the same test setups are used in this study. For the sake of completeness, both test setups are briefly described in this section.

### 5.1. In-Plane Fatigue Crack Setup for 2D Video

**Figure 5:**
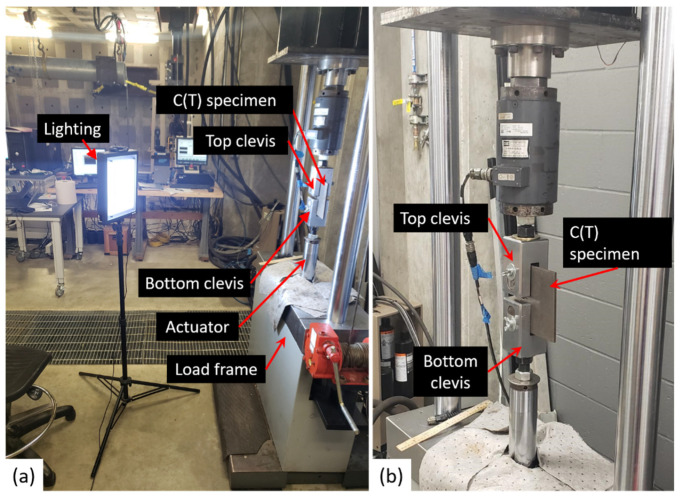
(**a**) Test setup for in-plane fatigue crack detection in a C(T) specimen, and (**b**) close-up view of the test setup.

A compact tension C(T) specimen was utilized to assess the effectiveness of the proposed crack detection algorithm in identifying in-plane fatigue cracks within 2D videos. The in-plane fatigue crack was generated by applying a uniaxial cyclic tension load with a servo-hydraulic load frame. The experimental setup is illustrated in Figure 5. The specimen was mounted in the load frame using two clevises. The loading protocol developed by Kong et al. [31], in accordance with ASTM E1820-15 [32], was employed to ensure realistic high-cycle fatigue loading. A fatigue crack was propagated under a loading frequency of 10 Hz and a stress ratio R of 0.6, maintaining a constant range of stress intensity factor ΔK = 22 MPa√m by progressively decreasing the load magnitude as the crack advanced. The C(T) specimen is made of A36 grade steel and has a thickness of 6.35 mm.

After the fatigue crack generation, a cyclical load varying between F_min_ = 2.67 kN and F_max_ = 6.23 kN at a frequency of 1 Hz was applied to the specimen. An 8 s video clip was captured utilizing a handheld Samsung Galaxy S9 Plus (Samsung Electronics Co., Ltd., Mountain View, CA, USA) smartphone. The footage was recorded in a resolution of 3840 × 2160 pixels at a rate of 30 frames per second. Additional lighting, as shown in Figure 5a, was employed during recording to counteract insufficient indoor illumination, which helped enhance the reliability of feature identification.

### 5.2. Out-of-Plane Fatigue Crack Setup for 3D Video

**Figure 6:**
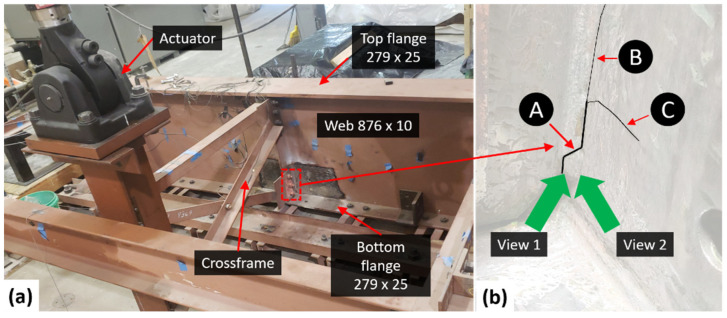
(**a**) Test setup for out-of-plane fatigue crack detection in a bridge girder specimen, and (**b**) close-up view of web-gap region and the fatigue crack.

Figure 6 illustrates the experimental setup for out-of-plane fatigue crack testing. Out-of-plane fatigue cracks frequently occur in the web-gap regions of steel girder bridges, where the girder web, flange, and crossframe intersect. A half-scale girder-to-crossframe subassemblage was utilized to validate the proposed approach for detecting out-of-plane fatigue cracks using 3D video recordings. The girder measures 2845 mm in length and 917 mm in depth, with a web thickness of 9.5 mm. Steel channels connecting the bottom flange of the steel girder are fixed to the laboratory strong floor, mimicking the real-world conditions of the top girder flange constrained by the concrete deck. A connection plate is used to attach a crossframe to a stiffener in the middle of the girder web. The connection plate is welded exclusively to the girder web to create the web-gap area, as depicted in the dashed box in Figure 6a. Applying a load at the far end of the crossframe imitates the traffic load, which leads to differential vertical displacement between neighboring girders and resulting in out-of-plane distortion-induced fatigue cracking in the web-gap region.

Figure 6b displays the fatigue crack with three unique branches, identified as A, B, and C. Initially, Branch A formed within the weld between the connection plate and web after 21,000 load cycles [33]. Subsequently, at approximately 1,700,000 cycles, Branch A continued to expand and split into two distinct branches, B and C, within the web [34]. However, the crack movement under fatigue loading was dominated by Branch A, as the propagation of the two branched cracks diminished the driving force at both crack fronts. As a result, Branch A was treated as the ground truth when assessing the detection accuracy. A 5 s video of resolution 1920 × 1080 pixels at a frame rate of 30 frames per second was captured using a head-mounted HL2. Similar to the 2D scenario, supplemental LED lighting was employed to compensate for the inadequate indoor lighting condition in the lab.

## 6. Quantification for Crack Detection

Multiple methodologies have been adopted for quantitatively evaluating outcomes in crack detection. Vision-based deep learning models for semantic segmentation assign a label to each pixel of the image. These pixel labels are then compared to the ground truth labels of the corresponding pixels. However, our vision-based approach uses salient feature points to analyze differential surface motion, and feature points surrounding the crack are highlighted as the detected crack. Hence, the crack detection result is composed of sparse pixel points rather than a continuous area labelled as the crack region. However, detecting the clusters of feature points for crack detection can be considered as segmenting the crack region based on the area associated with the detected feature points. Moreover, the width of the detected feature point cluster depends on the radius of the chosen LCR, which can be used to define the ground truth of crack region. To provide a quantitative measure of the crack detection result, a new approach is developed in this paper, which consists of three steps as follows: (1) clustering the detected feature points and forming boundaries of the clusters, (2) determining the boundary of the ground truth, and (3) evaluating the result based on the intersection over union (IOU). Details are explained as follows.

### 6.1. Clustering

Clustering involves organizing data points into groups according to their similarities. Within each group, data points exhibit a higher degree of similarity to each other than to data points in other groups. To determine the level of similarity or difference, a domain-specific dissimilarity measure (or distance metric) applicable to the dataset is utilized. Various algorithms for clustering exist, including K-means, Gaussian Mixture Models (GMM), Hierarchical Clustering, the Density-Based Spatial Clustering of Applications with Noise (DBSCAN), and Spectral Clustering. Among these, K-means and Hierarchical Clustering rely on distance for grouping, whereas DBSCAN focuses on the density of data points, and GMM is predicated on the mixture of Gaussian distributions. Both K-means and GMM necessitate predetermined input regarding the number of clusters, whereas Hierarchical Clustering provides a dendrogram that offers a range of clustering options based on different threshold levels. On the other hand, DBSCAN clusters the detected feature points based on density, and it produces a single result, forming clusters by connecting dense regions and treating noise as a separate group. Since it requires no manual input for number of clusters, DBSCAN was selected as the clustering method in this study. 

The DBSCAN algorithm depends on two principal parameters for its operation including the following: (1) epsilon (ε), which serves as a measure of neighborhood distance for determining cluster membership, and (2) the minimum number of points (MinPts), which establishes the threshold for cluster formation. Specifically, ε delineates whether a point is considered as a part of a cluster based on proximity. For instance, should a point be situated 13 units from cluster K and ε is set to 13 or more, the point is deemed a member of cluster K; if not, it is classified as external to the cluster K. MinPts, on the other hand, specifies the least number of points required to constitute a cluster. 

**Figure 7:**
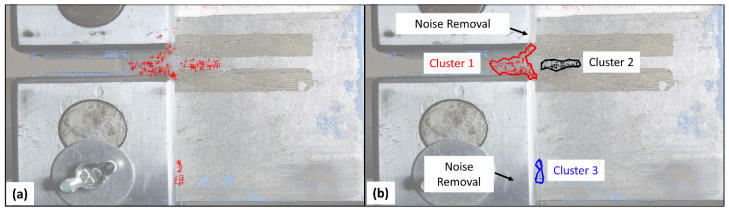
(**a**) Crack detection outcome using a low threshold value; (**b**) Clustering of the detected feature points on a C(T) specimen.

In this study, ε is defined as the radius of the LCR because our method relies on analyzing the movement patterns of sparse feature points within this radius. By setting ε equal to the radius of the LCR, we ensure that feature points located beyond this boundary are classified as belonging to a separate cluster. This distinction is crucial as feature points situated at greater distances should not be interpreted as part of the contiguous area identified as a crack region. In our study, the parameter MinPts is set to 10. This value is chosen to be sufficiently low to eliminate isolated feature points in the crack detection results. Setting MinPts to a value greater than 10 could erroneously classify a valid cluster of detected feature points as noise, which is not desirable. The DBSCAN methodology initiates with a random feature point, assessing its ε-neighboring points. This neighborhood qualifies as a cluster if the number of feature points is at least MinPts; if not, the point is tagged as noise. Subsequent points are then evaluated for potential inclusion in this or another cluster or identified as noise. To illustrate, Figure 7a presents the crack detection outcome using a low threshold value, while Figure 7b visualizes three distinct clusters colored in red, black, and blue. Feature points on the lower clevis and at the boundary with the plate, being more distant than ε from any cluster and numbering fewer than MinPts, are disregarded as noise. For identified clusters, boundaries are formed by connecting their outermost points, with every pixel within these boundaries classified as part of a crack. This process effectively transforms sparse feature points into continuous areas, providing a reliable basis for assessing accuracy of crack detection.

### 6.2. Ground Truth

**Figure 8:**
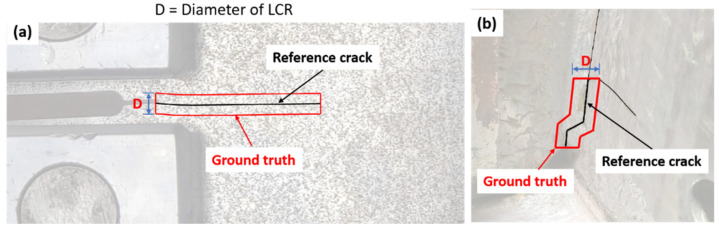
Ground truth labeling: (**a**) C(T) specimen; (**b**) bridge girder specimen.

The ground truth for crack detection is established by defining the actual dimensions and positioning of the cracks. Figure 8a illustrates the path of an in-plane fatigue crack, indicated by a continuous black line for the C(T) specimen. Given that this crack follows a straight trajectory, the ground truth is represented as a rectangular area, with its length corresponding to that of the crack. The width of the ground truth rectangle is determined to be the diameter D of the LCR since the width of the detected feature point cluster closely aligns with the LCR’s diameter. Meanwhile, the fatigue crack in the bridge girder specimen features three distinct branches, designated as A, B, and C. Due to the fact that Branch A dominated the crack movement, it is chosen as the reference for establishing the ground truth. As illustrated in Figure 8b, Branch A includes three linear sections; therefore, a polygon is formed to represent the ground truth. Similar to the C(T) specimen, the height of this polygon is defined based on the diameter D of the LCR.

### 6.3. Intersection over Union (IOU)

**Figure 9:**
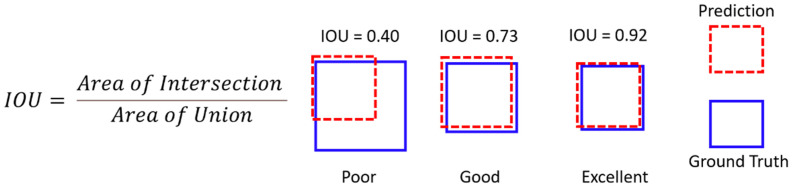
Illustration of different values of IOU.

With the clustering result and the ground truth, intersection over union (IOU) is computed as the metric for assessing the performance of crack detection. IOU describes the extent of overlap between two regions. The value of IOU ranges between 0 and 1, with 0 indicating no overlap and 1 indicating a perfect overlap. Figure 9 demonstrates examples of three different IOU values, in which an IOU score of 0.40 represents good performance in localization but not in coverage, an IOU score of 0.73 signifies satisfactory result in both localization and coverage, and an IOU of 0.92 exemplifies exceptionally high precision in both localization and coverage.

## 7. Crack Detection Results and Discussions

### 7.1. In-Plane Fatigue Crack Detection (2D Video)

**Figure 10:**
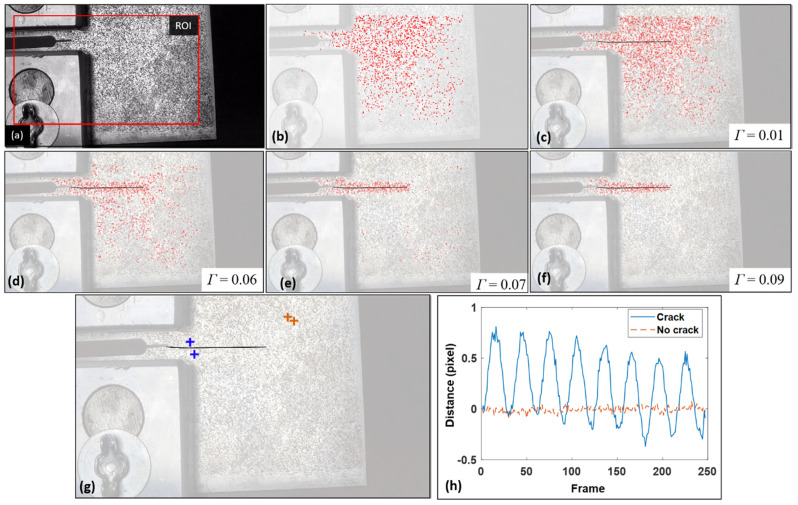
Crack detection using a 2D video: (**a**) The initial frame of the 2D video with the selected ROI; (**b**) all feature points detected by the Shi–Tomasi algorithm; (**c**–**f**) in-plane fatigue crack detection results under different threshold values; (**g**,**h**) locations and distance–time histories of feature point pairs for cracked and uncracked regions.

The proposed crack detection algorithm was applied to the recorded 2D video using the same parameters (ROI and LCR) used in Mojidra et al. [12], as depicted in Figure 10a,b. A total of 3000 feature points were detected using the Shi–Tomasi feature point detection algorithm. The diameter of the LCR depends on the camera resolution and the distance between structural surface and camera. In this study, a 35 pixel LCR radius was selected. More discussions on the impact of the radius of LCR can be found in Kong and Li [20]. Figure 10c–f present the crack detection results for various threshold values, with the reference crack marked by a black solid line as the reference. Figure 10c shows the result associated with a low threshold value of 0.01, which retains most of the feature points on the entire ROI. Figure 10d,e display results for increased threshold values of 0.06 and 0.07, respectively. However, they both still lead to feature points covering the entire ROI and are therefore not considered as the final crack detection results. Finally, when the threshold value was increased to 0.09, as shown in Figure 10f, the feature points were clustered in a local region and did not spread over the entire ROI, thus this is considered the final crack detection result. In field inspections, the inspector would examine these results and apply judgement to determine the optimal threshold value.

To illustrate the effectiveness of the proposed distance-based crack detection algorithm in identifying the motion pattern introduced by a crack opening and closing, distance histories of two feature point pairs from a cracked region and an uncracked region were extracted. A pair of feature points from the cracked region are represented by blue marks, while the other pair from the non-cracked region are highlighted by orange marks, as shown in Figure 10g. The distance–time history of the cracked region (Figure 10h) clearly shows eight cycles corresponding to the 0.5 Hz fatigue load applied to the C(T) specimen, leading to a higher variation in the motion pattern. On the other hand, the feature point pair from the non-cracked region has much lower distance change, resulting in a much lower motion variation. In the threshold scanning process, as the threshold value increases, feature point pairs with low relative differential motion will be filtered out, while feature point pairs with higher relative differential motion will cluster around the crack, hence these will be retained in the crack detection result.

**Figure 11:**
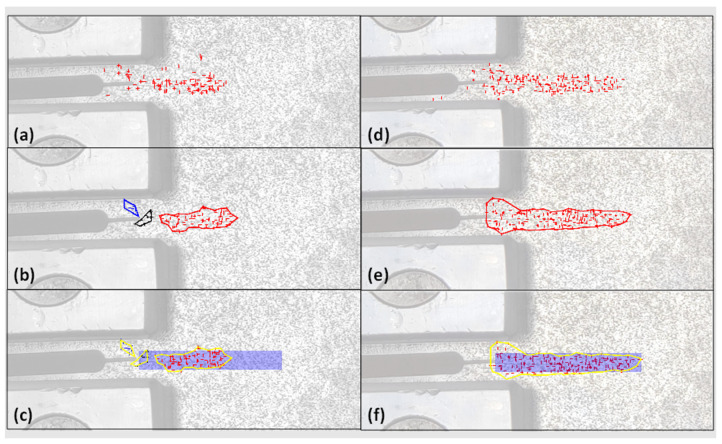
Quantification of crack detection in the C(T) specimen using the previous displacement-based method [12]: (**a**) detected crack by feature points; (**b**) clustering result; and (**c**) ground truth and the clustering result; and quantification of detected crack using the proposed distance-based method (this study): (**d**) detected crack by feature points (**e**) clustering result; and (**f**) ground truth and clustering result.

Once the fatigue crack was detected and localized, the accuracy of crack detection could be assessed using the developed quantification approach. First, clustering was performed on the selected crack detection result to convert the sparse feature points into continuous areas. For clustering, ε was set as 35 pixels, which was equal to the radius of LCR, and MinPts was set as 10. The clustered crack detection results for both the previous displacement-based method [12] and the proposed distance-based crack detection approach are compared in Figure 11b,e. As can be seen, a few isolated feature points in both methods have been eliminated as noise in the clustering results. However, the result of the distance-based approach formed only one cluster, while the result of the displacement-based approach formed three separate clusters. Finally, the IOU metric was computed for both crack detection results. In particular, the IOU for the displacement-based method was 0.38, while the IOU for the new distance-based approach was 0.73. For illustration, the clustering results were overlaid on top of the ground truth and are presented in Figure 11c,f. Evidently, the proposed distance-based approach achieved a significantly higher accuracy in fatigue crack detection in the 2D video.

### 7.2. Out-of-Plane Fatigue Crack Detection (3D Video)

**Figure 12:**
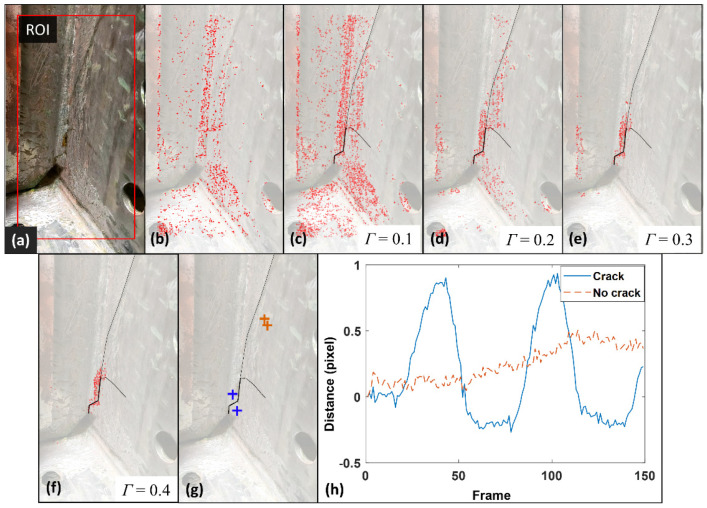
Crack detection using a 3D video: (**a**) The initial frame of the 3D video with ROI, all feature points detected by the Shi–Tomasi algorithm; (**b**–**f**) Out-of-plane fatigue crack detection results of the bridge girder specimen under various threshold values; (**g**,**h**) Location and distance–time histories of feature point pairs for cracked and uncracked region. Note that the brightness of the images in (**b**–**f**) is enhanced to highlight the feature points.

Figure 12a,b display the selected Region of Interest (ROI) and the initially detected features, respectively, aligning with the methodology of the displacement-based approach described in [12]. Utilizing the Shi–Tomasi feature point detection algorithm, a total of 5000 feature points were identified. Additionally, the radius of the LCR was set at 35 pixels. The outcomes of crack detection across various threshold values are depicted from Figure 12c–f, with the actual crack delineated in black lines serving as the benchmark. Figure 12c demonstrates crack detection employing a minimal threshold value of 0.1, leading to the retention of nearly all detected feature points. Figure 12d,e showcase the crack detection outcomes at elevated threshold values of 0.2 and 0.3, respectively. Despite this increment in threshold value, the feature points remained extensively scattered throughout the ROI, disqualifying these configurations from being considered as conclusive crack detection results. Conversely, Figure 12f illustrates the crack detection performance at a threshold value of 0.4, where the feature points are concentrated into a singular cluster, establishing this configuration as the definitive crack detection outcome.

The performance of the suggested distance-based approach for crack identification is highlighted by examining the temporal distance profiles of feature point pairs from both intact and damaged sections, as depicted in Figure 12g,h. A pair from a damaged area is marked in blue, while a pair from an undamaged section is indicated in orange. The distance trajectory of the pair in the undamaged area shows little to no variation over time, illustrating their uniform movement due to the camera’s rigid body motion. Conversely, the temporal distance profile of the pair in the damaged section prominently displays two distinct cycles with high amplitudes that mirror the external fatigue loading. These cyclic variations lead to a noticeable increase in standard deviation, which is key to detecting cracks by distinguishing differential surface movement patterns.

**Figure 13:**
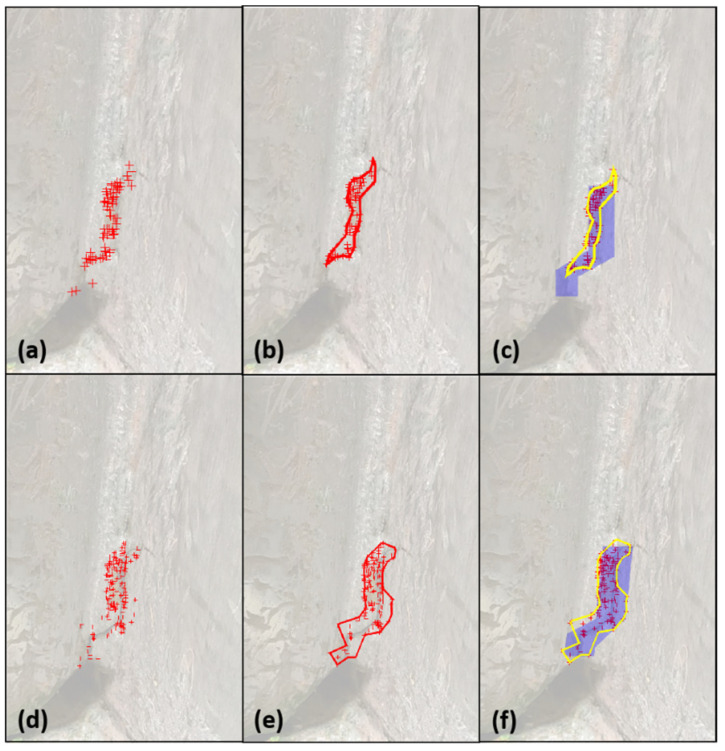
Quantification of crack detection in the bridge girder specimen using the previous displacement-based method [12]: (**a**) detected crack by feature points; (**b**) clustering result; and (**c**) ground truth and the clustering result; and quantification of detected crack using the distance-based method (this study): (**d**) detected crack by feature points; (**e**) clustering result; and (**f**) ground truth and clustering result.

Following the identification and localization of the fatigue crack, the accuracy of the detected crack was evaluated using both the displacement-based and the distance-based methodologies. The parameter ε was designated as 35 pixels, mirroring the radius of the LCR, while MinPts was established at 10. Based on these criteria, the crack detection outcome was consolidated into a single cluster, with several outlier feature points being discarded as noise. The cracks identified through the previous displacement-based method and the newly introduced distance-based strategy are depicted in Figure 13a,d, respectively. The clustering results are shown in Figure 13b,e. The IOU scores for the displacement-based and distance-based approaches are computed as 0.35 and 0.66, respectively. The clustered crack detection results are overlaid on the ground truth in Figure 13c,f. Consistent with the 2D video case, the proposed distance-based approach achieved significantly higher accuracy in fatigue crack detection in 3D video.

## 8. Parametric Study

Since the developed method relies on the crack opening and closing under live load, the result is significantly influenced by the load level. Therefore, a parametric study was carried out to examine the influence of fatigue load level on crack detection accuracy. Videos were captured from two distinct perspectives, as depicted in Figure 6b. View 1 observes the cracked region approximately equidistant from the connection plate and the girder web. In View 2, the video is recorded almost parallel to the girder web, resulting in a considerably greater parallax effect compared to View 1. A total of 10 fatigue load cases were considered in this parametric study as listed in Table 1. The minimum load level (Fmin) in each load case was 0.9 kN, and the maximum load level (Fmax) starts at 2.2 kN for LC1 and is increased by 2.2 kN in each subsequent load case. As a result, the first fatigue load case has a load range of 0.9 kN to 2.2 kN and the last fatigue load case ranges from 0.9 kN to 22.2 kN. In particular, based on a deflection criterion, the AASHTO fatigue truck loading [35] corresponds to an applied actuator load of 7.8 kN [34], making it somewhere between LC3 and LC4. The IOU values for the various load cases are tabulated in Table 1.

As shown in Table 1, results based on the videos recorded with View 1 angle have IOU values ranging from 0 to 0.77. The IOU scores can be categorized into two groups. The first group has lower IOU scores ranging from 0 to 0.40 for View 1 and from 0 to 0.44 for View 2. The second group has higher IOU scores ranging from 0.62 to 0.77 for View 1 and from 0.64 to 0.70 for View 2. As the load level increases, a corresponding increase in the IOU value is observed, enhancing crack detection capability. The IOU value is recorded at 0 for the initial load cases, LC1 and LC2, but increases to 0.34 and 0.40 for LC3 and LC4, respectively. For higher load cases from LC5 to LC10, IOU values exceed 0.62, indicating a significant overlap between the detected and actual crack areas, with more than two-thirds of the actual crack area being covered. In the context of View 2, the IOU values for LC2 to LC5 range between 0.42 and 0.44, with the detected crack area covering more than 64% of the actual crack area for load cases LC6 to LC10. Additionally, Table 1 includes the magnitude of crack opening for each load case, demonstrating the algorithm’s consistent performance in detecting fatigue cracks when the crack opening size surpasses 0.5 mm.

## 9. Validation of Human-Centered Bridge Inspection

**Figure 14:**
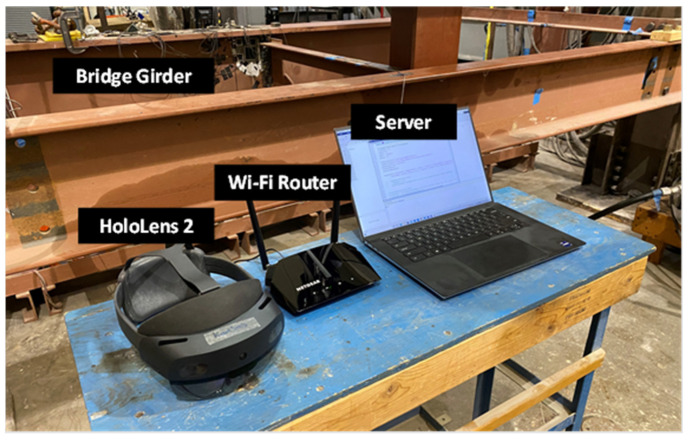
Experimental setup and hardware used in AR-based fatigue crack inspection.

**Figure 15:**
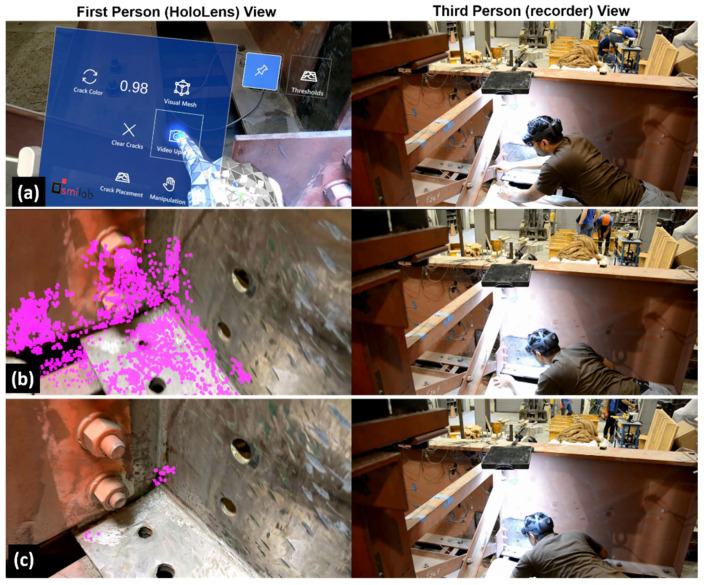
Demonstration of the integrated AR environment for human-centered bridge inspection: (**a**) Inspector starting the AR software, (**b**) inspector examining results for zero threshold, and (**c**) inspector examining the detected crack with the final threshold value.

The proposed human-centered bridge inspection approach was validated on the bridge girder specimen through the developed AR environment integrated with the proposed crack detection algorithm. First, as shown in Figure 14, the HL2 and the server, which hosts the MATLAB program for the crack detection algorithm, and the SQL database were both connected to a Wi-Fi hotspot. Then, the MATLAB program was initiated to enter the standby mode, waiting for a new inspection video to be uploaded to the database. The bridge inspector opened the AR software and adjusted the virtual menu to a convenient position using the flying menu button. Then, a 10 s video was recorded by pressing the video upload button as shown in Figure 15a, in which the recorder view of the figure shows the third-person view of the entire bridge inspection process, and the HoloLens view shows the first-person view of the bridge inspector through the HL2. Once the 10 s video was recorded, it was automatically sent to the server for crack detection using the MATLAB program.

Upon completion of the near-real-time processing of the video (approximately 30 s), the crack detection results corresponding to a range of threshold values were transmitted to the HL2. Subsequently, the AR software converted the feature points associated with the crack detection results into holograms and generated a virtual menu (Figure 4), from which the inspector could choose a specific threshold value to examine the crack detection outcome anchored on the bridge surface. For instance, as depicted in Figure 15b, the inspector initially selects the threshold value of zero to examine all the detected feature points from the analyzed region. This step provides the inspector with an understanding of the extent of the area assessed for fatigue crack detection. Subsequently, as illustrated in Figure 15c, the inspector selects a higher threshold value and evaluates the identified fatigue crack result. Throughout this process, the inspector altered its position multiple times and was able to see the fatigue crack feature points anchored on top of the crack, demonstrating the effectiveness of the automatic hologram anchoring system. This enhanced visualization aids the inspector in detecting and localizing fatigue cracks with minimal effort, making the inspection process more robust and efficient.

## 10. Computation Efficiency of the Distance-Based Algorithm

The elimination of GMC for camera movement adjustment significantly enhances the computational efficiency of the distance-based method over the displacement-based approach. On a system equipped with an Intel i9 processor and 64 GB of RAM, the distance-based method for analyzing a 2D video—comprising 248 frames at a standard HD resolution of 1980 × 1080 pixels—requires approximately 29 s, equating to a computational cost of around 0.12 s per frame. Meanwhile, the analysis of a 3D video, containing 149 frames of the same resolution, takes about 31 s on the same hardware, leading to a computation cost of roughly 0.20 s per frame. In contrast, the displacement-based method that incorporates GMC [12] demands significantly more computational time for both 2D and 3D videos, at 1.1 and 1.75 s per frame, respectively. A roughly 10-fold improvement in computational efficiency is achieved using the new method, which is the key to enabling near-real-time fatigue crack detection to facilitate the human-centered bridge inspection using AR and computer vision.

## 11. Conclusions

This paper presents an advanced methodology to enable human-centered bridge fatigue crack inspection by integrating computer vision and AR. A computer vision-based technique has been developed for fatigue crack detection utilizing video feature tracking from a moving camera. In addition, an AR environment has been created for HL2 to integrate the crack detection algorithm to enable near-real-time bridge inspection with human-in-the-loop decision-making. In particular, the proposed computer vision method relies on tracking surface motion through feature points and detecting and localizing fatigue cracks by examining differential movement patterns on the surface under fatigue loading within the video stream. One of the key contributions of this paper is addressing the challenge of near-real-time fatigue crack detection in videos recorded using a moving camera, which is critical for enabling near-real-time human-centered bridge inspection through AR. The differential motion pattern caused by fatigue crack opening and closing is determined by evaluating the changes in distance between feature point pairs. As feature points in close proximity experience the same motion under camera movement, assessing distance changes eliminates the need for GMC, resulting in near-real-time processing. In addition, a new method has been developed for quantifying fatigue crack detection results of the proposed algorithm based on the IOU metric. The crack detection results in terms of sparse feature points are clustered with the unsupervised DBSCAN algorithm, and then the outermost feature points are connected to convert the detection result into a continuous area to facilitate IOU computation. The proposed crack detection approach has been successfully validated in experimental setups using a compact C(T) specimen with an in-plane fatigue crack and a bridge girder crossframe subassemblage with a distortion-induced out-of-plane fatigue crack. Moreover, a parametric study has been performed to evaluate the impact of fatigue load level on the crack detection accuracy. Results show that the proposed crack detection approach gives satisfactory crack detection accuracy under the AASHTO fatigue load. The IOU scores and computation times suggest that the proposed distance-based approach outperforms the previous displacement-based method in both accuracy and computation efficiency. Finally, the developed AR environment consists of a cloud database hosted at the server for data communication, hologram generation from the crack detection result, an Auto Anchoring System for automatically anchoring the generated holograms onto the structural surface, and an interactive virtual menu to facilitate human–machine interaction during field inspections. The integrated AR environment has been tested on the laboratory bridge specimen which demonstrated its capability and potential for near-real-time human-centered bridge inspection in the field.

## Figures and Tables

**Table 1:** Parametric study of out-of-plane fatigue crack detection in the bridge girder specimen.

Load Case	Load Level (kN)		Crack Opening (mm)		IOU	
	F_min_	F_max_	View 1	View 2	View 1	View 2
LC1	0.9	2.2	0	0	0	0
LC2	0.9	4.4	<0.5	<0.5	0	0.42
LC3	0.9	6.7	0.6	0.6	0.34	0.44
LC4	0.9	8.9	0.7	0.72	0.40	0.43
LC5	0.9	11.1	0.9	1	0.72	0.44
LC6	0.9	13.3	1	1.2	0.68	0.68
LC7	0.9	15.6	1.1	1.5	0.77	0.64
LC8	0.9	17.8	1.3	1.64	0.66	0.68
LC9	0.9	20.0	1.5	2	0.71	0.70
LC10	0.9	22.2	1.8	2.2	0.62	0.69

## Data Availability

Some or all data, models, or code that support the findings of this study are available from the corresponding author upon reasonable request.
